# The Effects of Acute Exercise and Exercise Training on Plasma Homocysteine: A Meta-Analysis

**DOI:** 10.1371/journal.pone.0151653

**Published:** 2016-03-17

**Authors:** Rafael Deminice, Diogo Farias Ribeiro, Fernando Tadeu Trevisan Frajacomo

**Affiliations:** Department of Physical Education, State University of Londrina, Londrina-PR, Brazil; Idaho State University, UNITED STATES

## Abstract

**Background:**

Although studies have demonstrated that physical exercise alters homocysteine levels in the blood, meta-analyses of the effects of acute exercise and exercise training on homocysteine blood concentration have not been performed, especially regarding the duration and intensity of exercise, which could affect homocysteine levels differently.

**Objective:**

The aim of this meta-analysis was to ascertain the effects of acute exercise and exercise training on homocysteine levels in the blood.

**Method:**

A review was conducted according to the guidelines of the Preferred Reporting Items for Systematic Reviews and Meta-Analyses using the online databases PubMed, SPORTDiscus, and SciELO to identify relevant studies published through June 2015. Review Manager was used to calculate the effect size of acute exercise and exercise training using the change in Hcy plasmaserum concentration from baseline to post-acute exercise and trained vs. sedentary control groups, respectively. Weighted mean differences were calculated using random effect models.

**Results:**

Given the abundance of studies, acute exercise trials were divided into two subgroups according to exercise volume and intensity, whereas the effects of exercise training were analyzed together. Overall, 22 studies with a total of 520 participants indicated increased plasma homocysteine concentration after acute exercise (1.18 μmol/L, 95% CI: 0.71 to 1.65, *p* < .01). Results of a subgroup analysis indicated that either long-term exercise of low-to-moderate intensity (1.39 μmol/L, 95% CI: 0.9 to 1.89, *p* < .01) or short-term exercise of high intensity (0.83 μmol/L, 95% CI: 0.19 to 1.40, *p* < .01) elevated homocysteine levels in the blood. Increased homocysteine induced by exercise was significantly associated with volume of exercise, but not intensity. By contrast, resistance training reduced plasma homocysteine concentration (-1.53 μmol/L, 95% CI: -2.77 to -0.28, *p =* .02), though aerobic training did not. The cumulative results of the seven studies with a total of 230 participants in exercise training analysis did not demonstrate a significant impact on homocysteine levels in the blood (-0.56 μmol/L, 95% CI: -1.61 to 0.50, *p =* .23).

**Conclusions:**

Current evidence demonstrates that acute exercise increases homocysteine levels in the blood independent of exercise duration and intensity. Resistance, but not aerobic training decreases plasma homocysteine levels.

## Introduction

Homocysteine (Hcy) is a sulfur amino acid synthesized in the liver in response to methionine metabolism [1]. Hcy has recently gained attention in research due to its association with several diseases that can increase the risk of mortality [2–4]. In humans, a total Hcy level of 14.3 μmol/L or greater was independently associated with a relative risk of mortality, at rates of 54% for all-cause mortality and 52% for cardiovascular mortality [5, 6]. Humphrey et al. [7] demonstrated that each additional 5 μmol/L in Hcy levels increased the risk of cardiovascular events by approximately 20%. In fact, hyperhomocysteinemia decreases nitric oxide bioavailability and endothelial dysfunction [8], promotes the formation of toxic Hcy adducts (e.g., Hcy thiolactone), and favors oxidative stress, all of which can increase an individual’s susceptibility to atherosclerosis and thrombotic processes [9–11].

It is well established that physical exercise reduces the risk of cardiovascular disease [12, 13]. Studies have furthermore demonstrated exercise alters Hcy levels in the blood in rodents [14, 15] and humans [14, 16–18]. Recently, Silva Ade et al. [19] reviewed 34 studies and found generally higher levels of Hcy after acute exercise but no clear effect of exercise training. At the same time, Joubert et al. [20] reviewed nine cross-sectional studies and established no clear consensus regarding whether physical fitness affects Hcy levels. However, no meta-analyses of the effects of acute exercise and exercise training on Hcy concentration in the blood have been performed, especially regarding duration and intensity of exercise, which could impact Hcy levels differently.

Since exercise induces changes in Hcy levels, it is important to investigate whether acute exercise and chronic training affect Hcy levels in the blood. The growing number of published studies on related topics allows researchers to conduct analysis using rigorous methods and to summarize the primary results. Accordingly, we proposed to perform a meta-analysis in order to provide a statistical summary of comparable studies, chiefly as a means to consolidate a quantitative review of the effects of exercise on Hcy levels in the blood. We hypothesized that acute exercise increases Hcy concentration in the blood, especially long-term acute exercise, whereas regular exercise training down-regulates Hcy formation and decrease its levels in the blood.

## Methods

### Search approach and study selection

**Fig 1:**
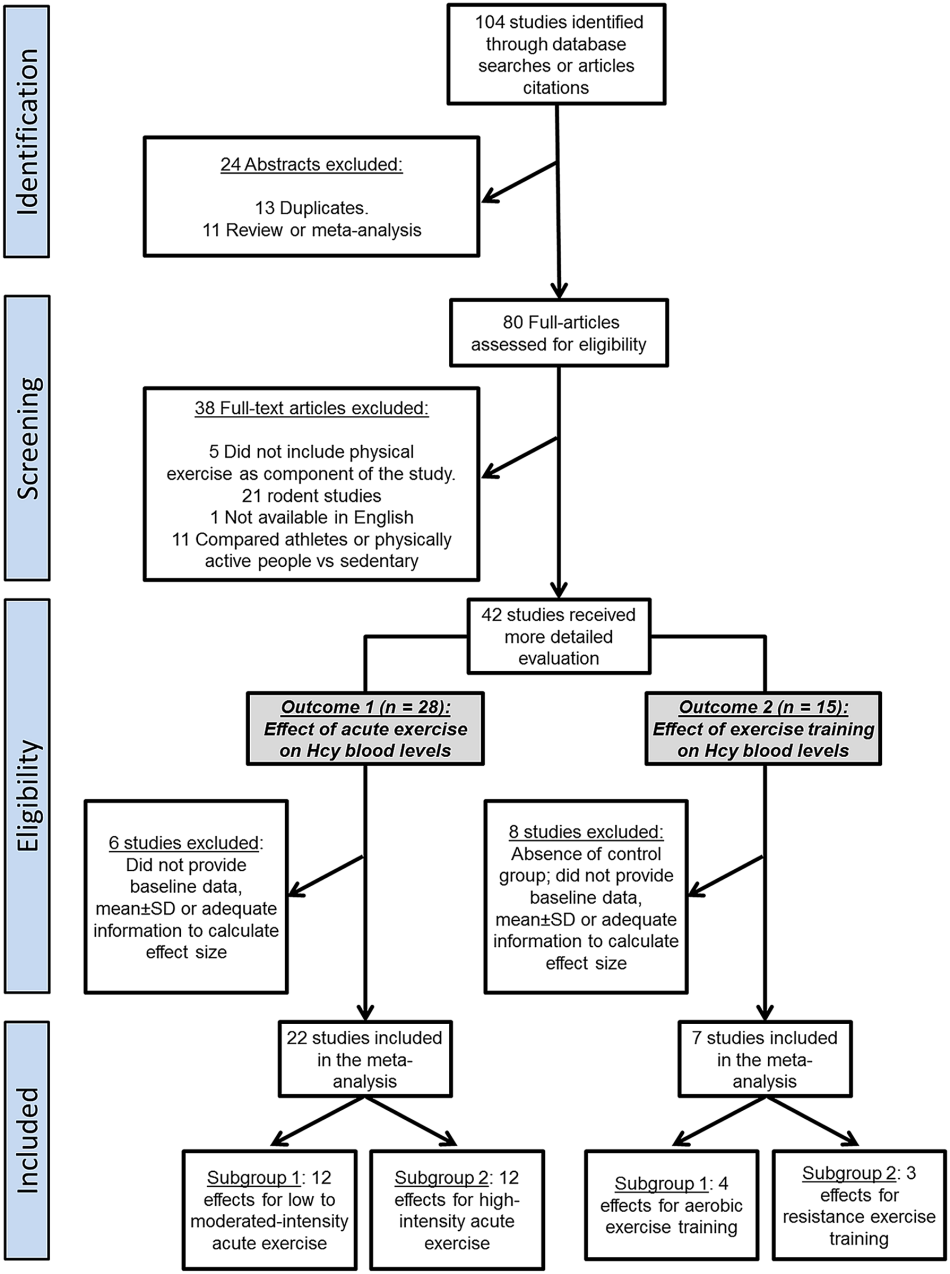
PRISMA flow diagram of the study selection process. After careful discussion between the 2 reviewers, two outcomes were identified and included in the meta-analysis.

This review was conducted in accordance with the guidelines of the Preferred Reporting Items for Systematic Reviews and Meta-Analyses [21, 22]. The online databases PubMed, SPORTDiscus, and SciELO were searched for English-language articles published through June 2015 using a combination of the terms “homocysteine,” “exercise,” “acute exercise,” and “exercise training,.” Any case–control, cross-sectional study, clinical trial, or cohort study in Hcy concentrations in humans in any exercise condition were analyzed first, after which the references of all review articles and original papers were examined and crosschecked. After the exclusion of duplicate publications, articles identified were included in the review, as long as they were available in English, involved human participants, and contained quantitative information regarding plasma or serum Hcy concentrations. Relevant articles cited in publications were carefully reviewed and included in the meta-analysis—again, as long as they matched those criteria. Records were excluded if they presented provided a review or meta-analysis, compared athletes or physically active people to sedentary people to identify Hcy concentrations in the blood instead of providing an exercise design, or had no resting control condition (i.e., pre-exercise values) or control group. The selection was not limited by study duration, exercise intensity, exercise modality, or initial fitness level of participants. Two authors (RD and DR) independently conducted the literature search using the same search strategy, which considered two primary outcomes: the effects of acute exercise on plasma–serum Hcy concentrations (Outcome 1) and the effects of exercise training on plasma–serum Hcy concentrations (Outcome 2). Ultimately, 104 articles were identified during the initial search process. The study selection process is described in Fig 1.

### Data extraction

The following data were extracted from articles included in the meta-analysis for Outcome 1: authors, year of publication, country of study, study design, number of participants, gender of participants, acute exercise intervention, sampling time, and other controls such as dietary records for B-vitamins intake, plasma volume changes to control hemoconcentration, and B-vitamins plasma or red blood cell (RBC) assay. In addition to those data, Outcome 2 also considered duration, frequency, and intensity of exercise training, study duration, and other controls such as dietary records for B-vitamins intake and plasma or RBC assay. All meta-analysis procedures were conducted as described by Stroup et al. [23].

Since exercise volume and intensity were not consistently reported by the studies, episodes of exercise were classified in terms of Outcome 1 as either long-term acute exercise of low-to-moderate intensity (effort <70% VO_2_max; <80% HRmax; >30 min of continuous exercise) or short-term acute exercise of high intensity (>70% VO_2_max; >80% HRmax; <20 min of intermittent or either exhaustive or progressive exercise). Using these criteria, a subgroup analysis was created to examine the effects of the relation of volume and intensity on plasma Hcy levels regarding Outcome 1. Due to variation among training interventions, a subgroup analysis was created in regard to Outcome 2: aerobic training and resistance training. If a study provided no information (i.e., qualitative assessment, VO_2_max, or heart hate values), then the exercise intervention was examined and a subjective determination made by the authors. For studies included in Outcome 1 that provided multiple time sampling points, the moment that presented the greatest difference (i.e., positive or negative) from the baseline was used.

### Data analyses

A meta-analysis of continuous outcomes was conducted using Review Manager (RevMan software package version 5.0), as were calculations of effect size of acute exercise (Outcome 1) and exercise training (Outcome 2), using the change in plasma-to-serum Hcy concentration from baseline to that following acute exercise and exercise training compared to concentrations in sedentary control groups, respectively. If the change from baseline data or corresponding *SD* were unavailable, then these values were calculated using standard statistical methods assuming a correlation of 0.50 between the baseline and post-intervention scores for each participant [24]. If studies reported standard error, then those values were converted to *SD*. For studies with non-parametric data reporting median and range, the equations of Hozo et al. [25] were used to estimate mean and *SD*.

Data from all included studies were used to calculate the weighted mean difference and 95% confidence interval (CI) using a continuous random effects model for both Outcomes 1 and 2. Weighted percentages were based on the sample sizes of respective studies. Statistical significance was assumed to be *p* < .05 in a *Z*-test analysis, which examined whether the effect size differed significantly from zero. Study heterogeneity was evaluated using the *I*^2^ statistic and Cochrane’s *Q*. Values of *I*^2^ greater than 50% and 75% were considered to indicate moderate and high heterogeneity, respectively. Significant heterogeneity was indicated when Cochrane’s *Q* exceeded the degrees of freedom (df) of the estimate. When meta-analysis was considered to indicate moderate-to-high heterogeneity and the random-effects model was used [26], publication bias was tested visually using a funnel plot. The dose–response relationship between volume and intensity of exercise and changes in effect size for plasma Hcy levels were determined using a bubble plot with the Pearson product-moment correlation coefficient.

## Results

### Literature search

The initial search returned 104 abstracts; Fig 1 illustrates how they were arranged. After duplicate studies, reviews, and meta-analyses were excluded (*n* = 24), 80 studies were assessed for their eligibility by applying the inclusion and exclusion criteria. As a result, another 38 studies were excluded; five studies did not include exercise as a component, 21 were studies with rodents, one study was unavailable in English, and 11 compared athletes or physically active people versus sedentary ones. Ultimately, 42 studies met the specific criteria proposed for the present meta-analysis (Fig 1).

### Effects of acute exercise (Outcome 1)

After extensive discussions between the authors, 28 articles were identified for inclusion in the meta-analysis for Outcome 1. Six of those studies were excluded, however, because they did not provide baseline (i.e., pre-exercise) data or adequate information to calculate effect size [27–32]. Consequently, only 22 studies were included in the meta-analysis for Outcome 1. Table 1 describes the characteristics of those studies.

**Table 1:** Characteristics of studies included in the acute exercise analysis (outcome 1).

Study	Country	Total (n)	Gender (M/F)	Participants characteristics	Acute exercise intervention	Sampling time	Other control
Murawaska-Cialowicz [42]	Poland	42	-	Athletes: acrobatic gymnastics (n = 6), judo (n = 8), biathlon (n = 8), rowing (n = 8) and road bicycle racing (n = 12).	CE Wingate test (judo and gymnastics) and progressive CE, TM or RE test (biathlon, rowing, road bicycle racing)	Before and 10 min after exercise	B6, B12 and folic acid serum assay
Hammouda et al. [54]	Tunisia	15	M	First division of the Tunisian football league.	Yo-Yo intermittent recovery test	Before and 3 min after the exercise.	Dietary records for B12 and folate intake and plasma volume changes control.
Deminice et al. [53]	Brazil	23	M	Under-20 soccer players	RAST twice with 2 min interval between.	Before, immediately after (post 0 h) and 1h after the exercise	Dietary records for B12 and folate intake, plasma volume changes control and B12 and folic acid plasma assay.
Saboorisarein et al. [43]	Iran	15	F	Healthy trained subjects	Bruce exercise test in the morning (8am) and evening (10pm)	Before and after exercise.	-
Iglesias-Gutiérrez et al. [33]	Spain	8	M	Young sedentary subjects	CE Isocaloric exercise at low intensity (40% VO2peak) and high intensity (80% VO2peak)	Before (4h and 0h), during (10min, 20min, 30min,45min, and 60 min), and after exercise (0h, 3h, and 19 h	Dietary records for B6, B12 and folate intake, and B6, B12 and folic acid plasma assay.
Hammouda et al. [34]	Tunisia	18	M	Young football players (~17 years of age)	CE Wingate maximum test	Before and 3-min after exercise.	Dietary records for B12 and folate intake and plasma volume changes control.
McAnulty et al. [44]	USA	25	M	Trained healthy subjects (~32 years of age)	TM running for 2.5 h at 72% VO2max	Before, immediately after and 1h after exercise.	Plasma volume changes control.
Bizheh [48]	Iran	14	M	Healthy and inactive adults	Resistance exercise session for ten exercises at ~35% 1-RM. Maximum repetition in 20s, for three sets with 1min rest interval.	Before and immediately after exercise	-
Benedini et al. [51]	Italy	5	M	Healthy marathon runners	Half Marathon	Before and after the marathon.	B12 and folic acid plasma assay.
Venta et al. [35]	Spain	29	M	Aerobic athletes Cyclists (n = 15) and kayakers (n = 14)	Incremental to exhaustion at CE for cyclists (~28min) and kayak ergometer for kayakers (~21 min)	Before and 30 ± 5 min after the exercise test.	Plasma volume changes control and B12 and folic acid plasma assay.
Subaşı et al. [47]	Turkey	38	M/F	Healthy sedentary students: resistance exercise group (n = 20); aerobic exercise group (n = 18)	Resistance exercise: 3x10-repetitions with 60s rest at ~80% 1-RM; Aerobic exercise: 30-min walking treadmill at 70–80% of HRmax.	Before and immediately after exercise.	-
Zinellu et al. [36]	Italy	16	M	Young subjects: sedentary (n = 6); Thai boxer athletes (n = 10)	Incremental CE test to exhaustion.	Before and after exercise.	-
Gelecek et al. [45]	Turkey	22	M/F	Sedentary healthy subjects	Walking treadmill for 30 min at 70–80% HRmax.	Before and immediately after exercise.	-
Sotgia et al. [37]	Italy	16	M	Young subjects: sedentary (n = 6); Thai boxer athletes (n = 10)	Incremental CE test to exhaustion.	Before and after exercise.	B12 and folic acid plasma assay.
Real et al. [52]	Spain	22	M	Non-professional marathon runners	Marathon race (42km)	Before and 24h after the marathon.	B12 and folic acid plasma assay.
Gaume et al. [38]	France	12	M	Middle-aged trained subjects (n = 12)	Trained subjects performed CE to exhaustion	Before, at submaximal, at exhaustion, and during recovery (2nd min and 15th min).	Dietary records for B6, B12 and folate intake
O’dochartaigh et al. [39]	Northern Ireland	10	M	Heathy young men	Incremental CE to exhaustion (duration ~13.2 min)	Before, during the peak of exercise and 4h after exercise.	-
König et al. [49]	Germany	39	M	Well-trained triathletes	Sprint triathlon (swimming—400 m, bicycle run– 25.000m, and run– 4.000 m) competition	Before and 1h and 24h after triathlon competition	B12 and folic acid plasma assay.
Herrmann et al. [50]	Germany	100	M/F	Recreational endurance athletes	Marathon race (n = 46); running 100 km (n = 12); mountain bike race 120 km (n = 42)	Before and 15 min and 3 h after the competition	B12 and folic acid plasma assay.
De Crée et al. [40]	UK	7	M	Moderately aerobically trained subjects	CE for 1h at 60% VO2max	Before and after exercise.	Plasma volume changes control.
De Crée et al. [41]	UK	15	F	Healthy young woman (~18 years of age)	Incremental CE test to exhaustion.	Before and after exercise at submaximal and maximal phase during the test; and 5 min and 10 min recovery	Dietary records for B6, B12 and folate intake and plasma volume changes control.
Wright et al. [46]	USA	20	M	Healthy, physically active subjects.	TMR for 30 min at 70% HRmax	Before, immediately and 30 min after exercise.	-

RE, rowing ergometer; CE, cycle ergometer; TMR, treadmill running; RAST, Running-based anaerobic sprint test; LI, Low Intensity; HI, High Intensity; HR, heart rate; HRmax, heart rate maximum; RM, repetition maximum; VO2max, maximal oxygen consumption.

In those studies, the sample size ranged from five to 100 participants per treatment. Many studies did not adequately report participant characteristics, but instead used general terms such as *healthy*, *active*, and *amateur athletes*. Ten studies used a cycle ergometer as the mode of acute exercise [33–42], four used treadmill running [43–46], two used resistance training [47, 48], four used a simulated or amateur competitive situation such as a half marathon or mountain biking course [49–52], and the remaining two compared specific protocols such as the running-based anaerobic sprint test [53] and Yo-Yo test [54]. Only six studies provided a controlled intensity parameter based on VO_2_max [40, 44] and HRmax [45–47]. Fourteen studies used only one sampling moment (i.e., after acute exercise) to measure plasma Hcy concentration, nine in the first 10 min after the exercise. Eight studies sampled multiple moments after acute exercise, including those that did not control any Hcy interferons.

**Fig 2:**
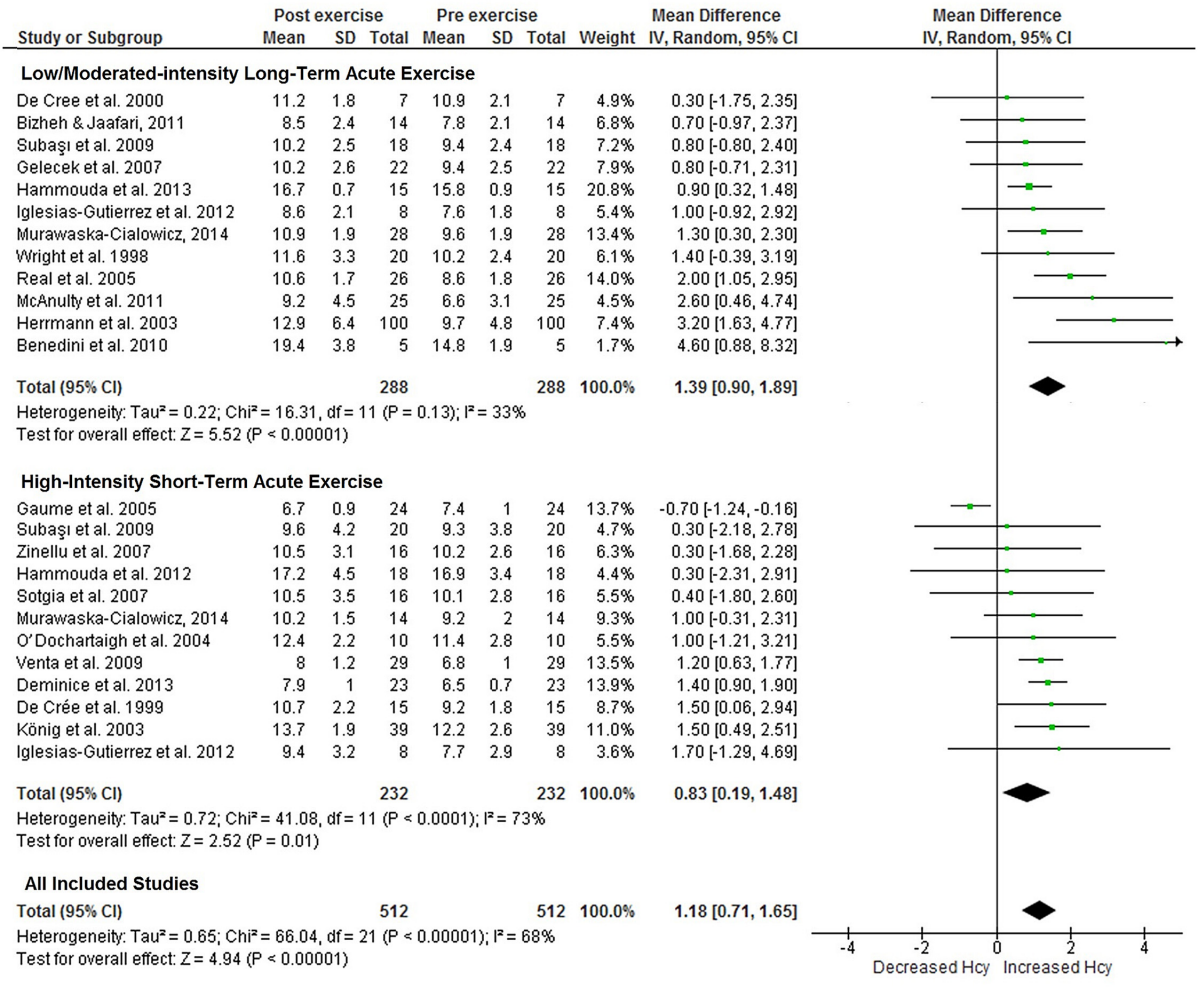
Meta-analysis performed on the effects of acute exercise on blood Hcy concentration demonstrated as the change in Hcy plasma/serum concentration from baseline to post-acute exercise. Calculation based on random effects model. Results are expressed as weighted mean difference (WMD) of Hcy (μmol/l) and 95% confidence intervals (95% CI).

The 22 studies included generated 12 effects in Subgroup 1 (i.e., long-term acute exercise of low-to-moderate intensity) and 12 effects in Subgroup 2 (i.e., short-term acute exercise of high intensity), with 232 and 288 participants, respectively. Only one of the 12 effects did not indicate increased plasma Hcy concentration after long-term acute exercise of low-to-moderate intensity. An increased mean effect size of 1.39 μmol/L (95% CI: 0.90 to 1.89, *p* < .01; Fig 2) with a low degree of heterogeneity (*I*^2^ = 33%; Cochrane’s *Q* = 16.3, df = 11, *p* = .13) was observed. Funnel plot inspection demonstrated symmetric distribution and an absence of publication bias. The 12 effects of Subgroup 2 agreed that short-term acute exercise of high intensity increases plasma Hcy concentration, with a mean effect size of 0.83 μmol/L (95% CI: 0.19 to 1.48, *p* < .01; Fig 2) and a moderate degree of heterogeneity (*I*^2^ = 73%; Cochrane’s *Q* = 41,08, df = 11, *p* < .01). For this subgroup, funnel plot inspection also demonstrated symmetric distribution and minimal publication bias.

Although both long-term acute exercises of low-to-moderate intensity and short-term acute exercise of high intensity demonstrated increased Hcy concentration after the exercise, changes in Hcy levels appear to be more sensitive to long-term exercises of low-to-moderate intensity, which elevate Hcy concentration to roughly 67% more than short-term, high-intensity exercise (Fig 2).

**Fig 3:**
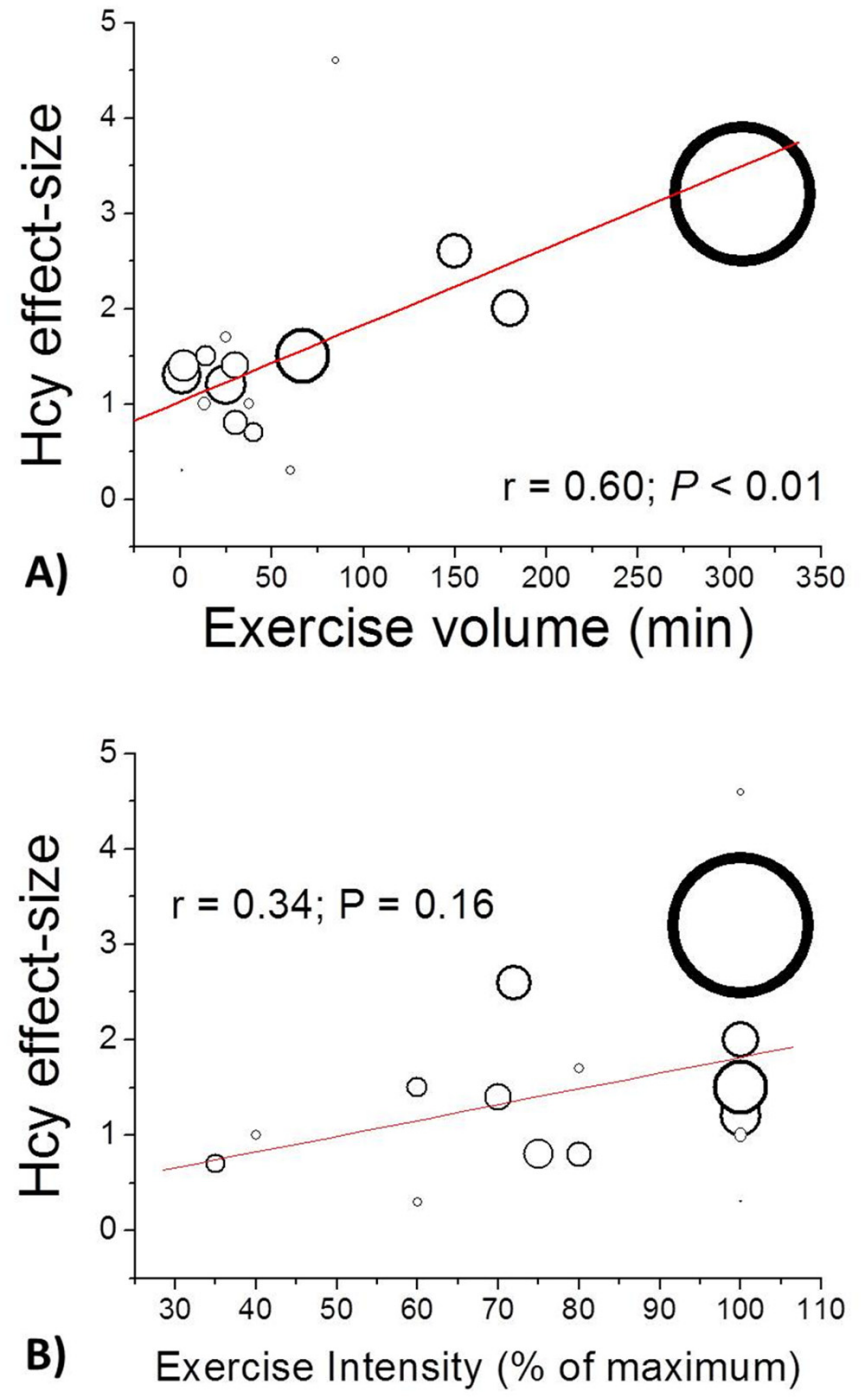
Bubble plot showing the dose–response relationship between the mean volume (A) and Intensity (B) of exercise intervention and effect size changes for Hcy plasma levels (%) for the eighteen included studies. For one single continuous variable, the fitted regression line together with circles representing the estimates from each study, sized according to precision of each estimate in the fitted random-effects meta-regression. Studies included: Murawaska-Cialowicz [42], Deminice et al. [53], Iglesias-Gutiérrez et al. [33], Hammouda et al. [34], McAnulty et al. [44], Bizheh [48], Benedini et al. [51], Venta et al. [35], Subaşı et al. [47], Gelecek et al. [45], Real et al. [52], O’dochartaigh et al. [39], König et al. [49], Herrmann et al. [50], De Crée et al. [40], De Crée et al. [41], Wright et al. [46].

When pooled, the 22 studies (i.e., 24 effects) selected for Outcome 1 included 520 participants and measurements of Hcy levels taken both before and after acute exercise. Only one of the 24 effects did not indicate increased mean Hcy concentration after acute exercise compared to the resting control before exercise. As Fig 2 shows, the cumulative results of the 24 effects gathered from the 22 studies included in Outcome 1 demonstrated plasma Hcy levels 1.18 μmol/L higher after acute exercise than before (95% CI: 0.71 to 1.65, *p* < .01), with moderated heterogeneity among studies (*I*^2^ = 68%; Cochrane’s *Q* = 66.04, df = 21, *p* < .01). Funnel plot inspection demonstrated symmetric distribution and minimal publication bias. As Fig 3 shows, we also noted a significant positive impact on Hcy effect size as exercise volume increased (*r* = .60, *p* < .01), which did not occur for Hcy effect size as exercise intensity increased (*r* = .34, *p* = .16).

### Chronic exercise (Outcome 2)

Eight of the 15 studies selected were excluded from outcome 2, either due to the absence of a control group or of baseline data, if not also adequate information for calculating effect size [28, 41, 55–58], as Fig 1 shows. Table 2 depicts the characteristics of the seven studies included in Outcome 2, which included a total of 260 participants; 153 submitted to an exercise training program, and 107 were sedentary individuals in the control group.

**Table 2:** Characteristics of studies included in the exercise training analysis (outcome 2).

Study	Groups (n) exercise/control	Gender (M/F)	Participants characteristics	Exercise training	Duration (min/d)	Frequency (d/wk)	Study length (wk)	Other controls
Antunes et al. [59]	22/23	45/0	Sedentary elderly subjects aged 60–75 years	AT on CE; HR monitoring	20 to 60 progressively	3	24	-
Bereket-Yucel [60]	20/20	40/0	Young university students	Resistance exercise training (3 sets of 6,8 and 10 RM for the first 4 weeks; 3 sets of 4,6 and 8 RM for the final 4 weeks	-	3	8	-
Gelecek et al. [45]	29/28	23/24	Sedentary healthy young subjects (21 years of age)	AT walking (6.4–7.0 km/h)	30 min	3	24	-
Vincent et al. [17]	29/20	-	Sedentary elderly subjects aged 60–72 years: Normal-weight (n = 20) Overweight/obese (n = 29)	Resistance Training: 8 to 13 repetitions of 13 muscle groups at 50 to 80% 1RM	-	3	24	-
Boreham et al. [61]	8/7	0/15	Sedentary young subjects	Progressive stair climbing programme from 1 to 5, climbing (199 steps) at 90 steps/min	-	5	8	-
Vincent et al. [18]	33/10	16/27	Healthy subjects 60–80 years	Resistance Training: 8 to 13 repetitions of 13 muscle groups at 50 or 80% 1RM	-	3	24	Dietary records for B6, B12 and folate intake and B12.
Randeva et al. [62]	12/9	0/21	Young, overweight or obese subjects with polycystic ovary syndrome	AT walking at self-select pace	20 to 60	3	24	Dietary records for B12 and folate intake and B12 and folic acid plasma assay.

AT, aerobic training; CE, cycle ergometry; HR, heart rate; 1RM, one-repetition maximum.

As also found for Outcome 1, many studies did not adequately report participant characteristics. Three studies included elderly people aged more than 60 years [17, 18, 59], whereas others included healthy young people [45, 60, 61] and obese individuals [62]. The exercise training interventions varied in design and included aerobic exercises such as walking [45, 62], step climbing [61], and cycle-ergometer [59] training programs, as well as resistance training programs [17, 18]. The three resistance training studies used one-repetition maximum as the intensity parameter. As shown in Table 2, only one of the three aerobic training studies assigned exercise intensity as a percentage of maximal heart rate [59] (Table 2); the others used subjective parameters. Duration of exercise ranged from 20 to 60 min, though four studies did not indicate duration. Most studies had an exercise training frequency of 3 d per week for 24 weeks. Only two of the seven studies controlled for b-vitamin intake or blood levels, if not both [18, 62].

**Fig 4:**
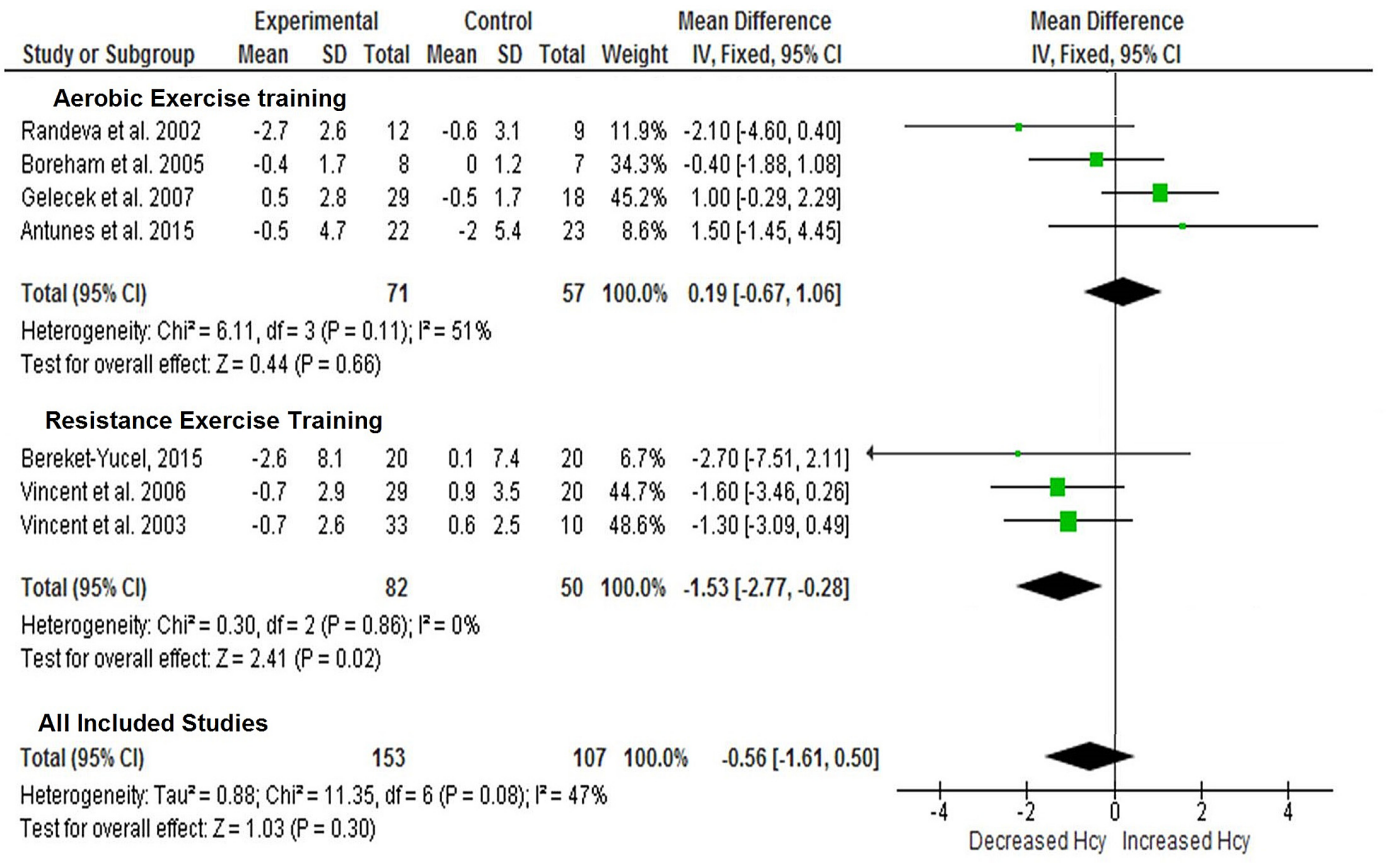
Meta-analysis performed on the effects of exercise training on blood Hcy concentration demonstrated as trained vs sedentary control groups. Calculation based on random effects model. Results are expressed as weighted mean difference (WMD) of Hcy (μmol/l) and 95% confidence intervals (95% CI).

As shown in Fig 4, subgroup analysis demonstrated that resistance training reduced plasma Hcy concentration (-1.53 μmol/L, 95% CI: -2.77 to -0.28, *p =* .02), which aerobic training did not (0.19 μmol/L, 95% CI: -0.67 to 1.06, *p =* .66). Fig 4 also indicates that the cumulative results of the seven studies included in the analysis did not demonstrate any significant impact on Hcy levels in the blood (-0.56 μmol/L; 95% CI: -1.61–0.50, *p =* .30). For this outcome, the *p* value for the heterogeneity test was significant (*p* = .08), as consistent with a previous analysis of acute studies. However, the *I*^*2*^ was low (*I*^*2*^ = 47%). These data indicate low diversity across studies regarding methodological aspects. Funnel plot inspection demonstrated symmetric distribution and minimal publication bias.

## Discussion

The present study sought to investigate whether acute exercise and exercise training influence Hcy levels in the blood. Overall, analysis showed that acute exercise increases Hcy levels independent of exercise intensity. Whereas resistance training was found to decrease plasma Hcy levels, aerobic training had no effect.

Analysis of acute exercise (Outcome 1) revealed that in 21 of the 22 studies included in the meta-analysis, the mean Hcy concentrations were greater after acute exercise than before. As shown in Fig 2, the magnitude of the Hcy plasma increase was 1.18 μmol/L (~14%). This finding is particularly relevant considering the heterogeneity of the studies regarding the exercise protocol, exercise volume and intensity, age of participants, and their initial fitness, among other factors (Table 1). Data from the present study corroborate those of a previous review study [19], which reported that Hcy levels increased after acute physical effort, whereas Joubert et al. [20] observed mixed results in response to acute exercise. It is worth noting, however, that the increase in Hcy after acute exercise is transitory and liable to return to baseline levels in less than 24 h independent of exercise intensity [33]. In addition, increased Hcy induced by acute exercise (<1.2 μmol/L; <15%) was slight compared to that caused by pathological conditions such as renal chronic failure [63], dementias [64, 65], and cardiovascular disease [66]. Therefore, hyperhomocysteinemia (>15 μmol/L) caused by acute exercise remains unusual, for very few participants presented hyperhomocysteinemia after acute exercise [50]. Indeed, increased Hcy induced by acute exercise might not be deemed a risk factor of cardiovascular events mediated by hyperhomocysteinemia [7].

Exercise variables such as intensity, volume, and duration can significantly influence the response of Hcy formation to acute exercise [49, 50]. Considering exercise volume and intensity stratification criteria in the present study, alterations in Hcy promoted by acute exercise were independent of intensity and volume. Although greater after long-term acute exercise of low-to-moderate intensity, a significant increased mean Hcy effect size was observed with both long-term exercise of low-to-moderate intensity (1.39 μmol/L; 16%) and short-term exercise of high intensity (0.83 μmol/L; 8%). As exercise volume increased, a significant positive impact on Hcy effect size was noted, though not as intensity increased (Fig 3). Running a marathon produced the most important acute Hcy elevations compared to that of other long-distance, well-trained endurance athletes [50]. Lower training volume (9.1-h training/week) also induced a significant rise in plasma Hcy 1 h after intensive competitive exercise and remained higher 24 h after than for athletes performing a high volume of exercise (14.9-h training/week) [49]. However, such variables are not well controlled for in most of studies included in the analysis (Table 1), despite the need to elucidate whether intensity, duration, or volume determine Hcy alterations during acute exercise.

Although the majority of studies demonstrated elevated plasma Hcy concentrations after acute exercise, the mechanistic explanation for this effect remains poorly investigated. Increased protein catabolism caused by exercise has been hypothesized by several studies to play a causative role in increasing plasma Hcy concentration. Since exercise increases the pool of amino acids in the muscles [67], it could also increase protein turnover and the catabolism of the intermediary metabolism for Hcy formation [20, 68]. Studies have shown increased plasma and muscle-free amino acids after acute exercise [16, 35, 67], which can also reduce glycogen reserves that in turn increase the demand for vitamin B-6 and folate [50] required for Hcy catabolism and removal, respectively.

Recent studies have additionally suggested that exercise can increase methyl flux and thus Hcy formation. Exercise clearly increases the demand for several methylated compounds such as DNA, epinephrine, acetylcholine, carnitine, and creatine [16, 53], which along with Hcy are products of transmethylation reactions. Sotgia et al. [37] demonstrated that variations in Hcy concentration induced by exercise are related to changes in the concentration of plasma creatine, an important methylated compound. However, the exact mechanism by which acute exercise increases Hcy levels remains unknown.

The cumulative results of the seven studies included in Outcome 2 revealed that regular exercise training does not reduce plasma Hcy concentration compared to levels in sedentary control individuals. Only three of the seven studies included demonstrated reduced Hcy levels in the blood after exercise training, all of which used resistance training as the exercise intervention [17, 18, 60]. Based on this evidence, we decided to conduct the meta-analysis in two subgroups: one with a resistance training regimen, the other with an aerobic training regimen. When subgroups were analyzed, resistance training was shown to decrease plasma Hcy levels from baseline, though aerobic training was not (Fig 4). This result is in partial accordance with our initial hypothesis that exercise training could decrease Hcy levels in the blood, particularly considering the acute changes promoted by exercise that in turn promote metabolic adaptation.

However, our exercise training analysis needs to be interpreted with caution. Most studies analyzed for initial inclusion in Outcome 2 did not include a sedentary control group, which significantly decreased the number of studies in the analysis. In addition, most studies included did not control exercise intensity or volume, especially in those addressing aerobic training. Differences in the modalities of exercise furthermore characterized the studies (Table 2). These facts could contribute to the non-differences found in overall and subgroup analysis for aerobic training.

The low number of studies included in Outcome 2 appears to be the major limitation of the present study. The decreased Hcy levels in the blood demonstrated in studies of resistance training appear to relate to the increased control of volume, intensity, and movement execution compared to walking and/or stair-climbing activities, for example [61]. Indeed, a lack of studies that controlled exercise volume and intensity, as well as their absence of control groups, could have masked the real effect of exercise training on plasma Hcy levels.

## Conclusion

Altogether, the results of the present meta-analysis provide evidence that acute exercise increases plasma Hcy concentration independently of duration or intensity of the exercise performed. However, Hcy elevation induced by acute exercise may not cause hyperhomocysteinemia and cannot be associated with an increased risk of developing cardiovascular disease. At the same time, regular resistance training can decrease plasma Hcy concentration, though such was not observed after aerobic exercise training. Future investigations should be aware of the need to better control exercise variables such as duration and intensity. Kinetic studies that include multiple sampling points regarding Hcy metabolism are recommended in order to clarify mechanisms Hcy metabolism in regard to exercise.

## Supporting Information

S1 PRISMA Checklist PRISMA meta-analysis checklist. (PDF)
